# Self-Powered UV Photodetector Construction of the P(EDOS-TTh) Copolymer-Modified ZnO Nanoarray

**DOI:** 10.3390/nano14080720

**Published:** 2024-04-20

**Authors:** Aygul Kadir, Tursun Abdiryim, Xiong Liu, Ruxangul Jamal, Yaolong Zhang

**Affiliations:** 1 State Key Laboratory of Chemistry and Utilization of Carbon Based Energy Resources, Key Laboratory of Advanced Functional Materials, College of Chemistry, Xinjiang University, Urumqi 830046, China; aygul419@sina.com (A.K.); liuxiong@xju.edu.cn (X.L.); zyl1754915507@sina.com (Y.Z.); 2 Key Laboratory of Petroleum and Gas Fine Chemicals, Educational Ministry of China, College of Chemical Engineering, Xinjiang University, Urumqi 830046, China; jruxangul@xju.edu.cn

**Keywords:** poly(3,4-ethylenedioxyselenphene), ZnO nanoarray, poly(terthienyl), self-powered UV photodetector

## Abstract

To solve the problem that zinc oxide nanorods (ZnO NRs)-based self-powered ultraviolet (UV) photodetectors cannot obtain both higher responsiveness and shorter response time, P(EDOS-TTh) was prepared using 3,4-ethylenedioxyselenphene (EDOS) and terthiophene (TTh) as copolymers, which modify the ZnO NRs surface, and the ZnO/P(EDOS-TTh) P-N junction self-powered UV device is assembled. The effect of the number of electrochemical polymerization cycles on the UV photodetection performance of ZnO/P(EDOS-TTh) P-N heterojunction was studied by adjusting the number of electrochemical polymerization cycles at the monomer molar ratio of 1:1. Benefiting from the enhanced built-in electric field of the ZnO/P(EDOS-TTh) interface, balancing photogenerated carriers, and charge separation and transport. The results show that the contact between N-type ZnO NRs and P-type P(EDOS-TTh) is best when the number of polymerization cycles is 3, due to the fact that EDOS-TTh and ZnO NRs form excellent P-N heterojunctions with strong internal electric fields, and the devices show good pyroelectric effect and UV photodetection performance. Under 0 V bias and 0.32 mW/cm^2^ UV irradiation, the responsivity (R) of ZnO/P(EDOS-TTh) reaches 3.31 mA/W, the detectivity (D*) is 7.25 × 10^10^ Jones, and the response time is significantly shortened. The rise time is 0.086 s, which exhibited excellent photoelectric properties and stability. UV photodetection performance with high sensitivity and fast response time is achieved.

## 1. Introduction

Ultraviolet (UV) photodetectors have attracted much attention in the past decades, owing to their use in applications such as fire alarms, missile tracking, ozone detection, communications, environmental monitoring, and image sensing [1,2,3,4,5].

1D ZnO nanostructures have gained growing interest owing to their high efficiency and potential applications in nano-optoelectronic devices such as ultraviolet photodetectors, photovoltaic cells, light-emitting diodes, and nanoscale lasers [6,7,8,9]. ZnO nanostructures (nanorods, nanoparticles, nanowalls, and nanowires) are one of the ideal materials for constructing UV photodetectors due to their superior responsiveness and fast recovery time compared to thin films or block-based photodetectors [10,11,12,13]. However, most ZnO-based photodetectors are driven by external bias, which greatly limits their independence in practical applications. In addition, the response speed of the device tends to be reduced due to local disturbances in the device depletion region caused by external power sources [14]. In recent years, ZnO-based photodetectors with self-powered characteristics have received more and more attention because they do not require any bias voltage to drive the device [15,16,17]. There are two main types of photodetectors, one of which is to use the photovoltaic effect and introduce a built-in electric field into the device through a p-n junction or Schottky junction. The other is the integration of optical sensors or storage units with energy-harvesting capabilities [18]. Among them, the p-n type UV photodetectors have the advantage compared to the Schottky junction device due to the effective separation of the generated carrier. ZnO NRs/organic P-N junction devices not only have the high intrinsic carrier separation of the ZnO NRs but also have the advantages of excellent hole transport for organic materials.

In recent years, as novel p-type materials, copolymers have been applied in solar cells and optoelectronic devices. Hu et al. [19] designed and synthesized a random copolymer consisting of naphthalene diimide (NDI), diketopyrrolopyrrole (DPP), and thiophene (T). By increasing the DPP content, the band gap of the copolymer decreased significantly. Moreover, the film morphology significantly improved, and the dark current of the photodetector significantly decreased. Among the PNDI-DPP10 formulations with a DPP/NDI ratio of 10/90, the molecular orientation and film morphology were the best. At the −0.1 V bias voltage, the detectability of the photodetector reached above 10^12^ Jones. Tong et al. [20] designed and fabricated a bulk heterojunction (BHJ) photodetector. The organic BHJ active layer was composed of [6,6]-phenyl C61 butyrate methyl (PCBM) and a donor–acceptor (PBDTT-ffQx) copolymer because the energy level matching between PBDTT-ffQx and PCBM can effectively separate the photogenerated electron-hole pairs, thus improving the properties of the photodetectors. Li et al. [21] studied the effect of the structure of conjugated polymers on the performance of photodetectors. They prepared four donor–acceptor-conjugated polymers and applied them to photodetectors. The studies showed that differences in the structural units led to differences in detection performance; for example, the highest D* of the device was 6.2 × 10^12^ Jones, and the best response time was 160 µs. Pickett et al. [22] designed the two copolymers, PBDT-TTDPP and PBDT-TDPP, which are built into photodetectors. The introduction of DPP enhanced the carrier transport and separation of the PBDT-TTDPP copolymer. Compared with PBDT-TTDPP:ZnO, the response of PBDT-TDPP:ZnO was improved by nearly two orders of magnitude. EDOS and thiophene as copolymerization monomers for copolymers can combine the advantages of EDOS and thiophene to make copolymers with higher hole transport efficiency.

In this paper, EDOS and terthiophene (TTh) were used as comonomers to deposit a copolymer (P(EDOS-TTh)) membrane on the surface of FTO conductive glass by an electrochemical method, and a ZnO/P(EDOT-TTh) heterojunction UV photodetector was assembled by way of face-to-face. The prepared materials morphology, structure, and optical performances of the ZnO/P(EDOTTTh) heterojunction composite were systematically studied to investigate the effect of copolymers with different numbers of rings on the properties of ZnO-based devices. Since a P-N junction can be constructed between n-type ZnO NRs and p-type P(EDOS-TTh) to generate a good built-in electric field, an as-prepared ZnO/P(EDOS-TTh) P-N heterojunction UV photodetector is projected to realize excellent performance self-powered UV photodetection.

## 2. Experimental Methods

### 2.1. Experimental Reagents

Butane-2,3-dione and trimethylformate were obtained from J&K Scientific Co., Ltd. (Beijing, China). Selenium was obtained from Sigma-Aldrich (Shanghai, China). tert-butyl hexafluorophosphate (TBAPF6), zinc acetate, zinc nitrate, and tertiary thiophene (Aladdin, Shanghai, China).

### 2.2. Materials Structure and Photoelectric Measurement

UV-visible spectra were measured for optical information by a Unico UV 2550 spectrophotometer. The all-material crystalline structure characterization was analyzed by X-ray diffraction (XRD) carried out on a Bruker AXS D8 diffractometer in the range of 10~80°. The functional groups of the composite were measured by BRUKER-QEUINOX-55 FTIR. The scanning electron microscope (SEM) was acquired using a SO8010 (Japan). X-ray spectroscopy (EDS) was used to determine the surface morphological characteristics and elemental distribution of the materials. The element chemical state information was obtained by high-resolution X-ray photoelectron spectra (XPS) taken in ESCALAB 250Xi.

### 2.3. Preparation of ZnO NRs and P(EDOS-TTh)

According to previously reported literature [14,15], ZnO NRs were grown on FTO-conducting glass by the hydrothermal method. The fluorine-doped tin oxide (FTO) glass substrates were initially ultrasonic cleaned with acetone, ethanol, and deionized water, successively, and then blown dry with dry air. The ZnO seed layer was first deposited on the fluorine-doped tin oxide (FTO) glass by the spin-coating process. Preparation of zinc oxide solvent: dissolve 0.5488 g zinc dihydroacetate in 50 mL ethanol. At 60 °C, the amount of eththanolamine (about 0.15 mL) with zinc is added to the above solution and stirred for 2 h to stabilize the transparent solvent. An aqueous solution for ZnO growth was prepared with 25 mM hexamethylenetetramine and 25 mM zinc nitrate. A piece of FTO substrate with the ZnO seed layer was placed at an angle against the wall of the autoclave, with the conducting side facing down. The autoclave was sealed and placed in an oven at 95 °C for 4 h before the sample was rinsed with deionized water.

**Figure 1:**
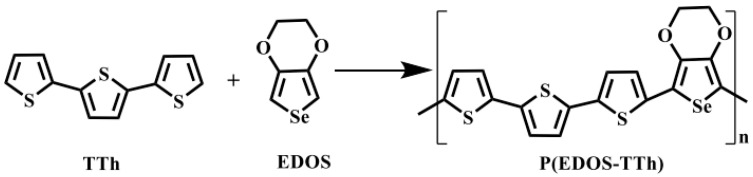
The route of electrochemical synthesis of P(EDOS-TTh).

The copolymer P(EDOS-TTh) membrane was prepared as follows [23]: EDOS and TTh (containing 0.01 M) were used as comonomers at a ratio of 1:1, and 0.05 M tetrabutylammonium perchlorate (TBAP) was for the electrolyte. Acetonitrile and dichloromethane at a solvent ratio of 7:2 were used as solvents. The electrochemical deposition was performed by cyclic voltammetry (CV) in the range of +1.5~−1.5 V under a scan rate of 100 mV/s. FTO glass was used as the working electrode, a Pt electrode was applied as the counter electrode, and an Ag/AgCl electrode was used as the reference electrode, respectively, throughout the electrochemical polymerization process. Figure 1 shows the road diagram for the electrochemical preparation of P(EDOS-TTh). Finally, the prepared materials were washed by dichloromethane and dried naturally to obtain a bright brown-red copolymer P(EDOS-TTh) membrane. Moreover, copolymer membranes with different numbers of polymerization cycles (from 1 to 5) were fabricated with the controlling number of polymerization cycles.

### 2.4. Preparation of the ZnO/P(EDOS-TTh) Heterojunction UV Photodetector

**Figure 2:**
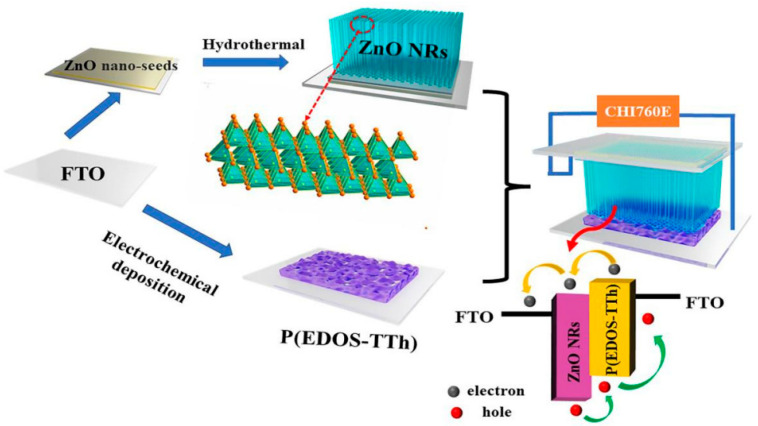
Schematic diagram of nanocomposite fabrication and assembly of the device.

Figure 2 shows a schematic diagram of the fabrication route of the ZnO/P(EDOS-TTh) composite and the assembly process of the P-N heterostructure device. FTO grown with P(EDOS-TTh) copolymer films with different numbers of polymerization cycles was applied as the working electrode, and another FTO grown with ZnO NRs (the same size) was applied as the counter electrode. Then, the P(EDOS-TTh) copolymer surface and the ZnO NRs/FTO glass were attached face-to-face to form a ZnO/P(EDOS-TTh) organic-inorganic P-N junction, and the illumination area of the device was 0.24 cm^2^. In this work, the photoelectric performance test was performed by an electrochemical workstation (CHI 760E) and a xenon lamp (CHF-XM500).

## 3. Results and Discussion

### 3.1. Structural Characterization of the As-Prepared Materials

**Figure 3:**
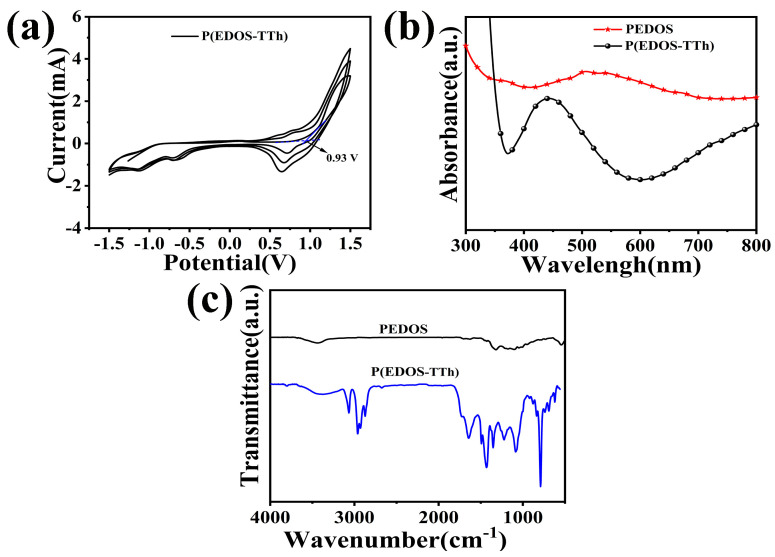
(**a**) CV curves of electrochemical polymerization of the P(EDOS-TTh) monomer. (**b**) UV-vis. (**c**) FT-IR spectra of materials.

Figure 3a shows the cyclic voltammetry curve of the electrochemical polymerization of the copolymer P(EDOS-TTh) on FTO glass. It can be seen from the figure that when the ratio of the monomer EDOS to TTh was 1:1, the initial oxidation potential of the P(EDOS-TTh) was 0.93 V, and the redox current increased as the number of polymerization cycles increased from 1 to 5, indicating the success of the copolymer film preparation [24].

Figure 3b shows the UV-vis of PEDOS and copolymer. PEDOS has a wide peak at 450~620 nm and an absorption peak center at 550 m, which is attributed to the π-π* transition of electrons in the PEDOS main chain. For the P(EDOS-TTh) copolymer, there was a broad absorbance in the range of 380–550 nm, and the center of the peak was located at 450 nm. Compared with that of PEDOS, the UV-vis absorption peak of the P(EDOS-TTh) copolymer was stronger, which was due to the characteristic structure of the copolymer. This absorption exhibited by the copolymer is beneficial for enhancing the optoelectronic properties of the hybrid-structure heterojunction device.

Figure 3c illustrates the FT-IR of PEDOS and P(EDOS-TTh), in which the peaks located at 689 and 793 cm^−1^ are attributed to the C-H bond in the thiophene, and the two peaks located at 1495 and 1590 cm^−1^ are contributed to the -C-C- bond on the thiophene ring and stretching vibration of -C=C- [23]. The peaks at 673 and 828 cm^−1^ are assigned to the stretching vibration of C-Se in the selenophene ring. The stretching vibrations of ethylene oxide at 1180, 1049, and 1288 cm^−1^ contribute to the C-O-C stretching vibration. The characteristic peaks at 1301 and 1501 cm^−1^ are attributed to the stretching vibrations of C=C and C-C on the selenophene ring in the PEDOS [25].

### 3.2. Morphology Analysis of the Copolymer

**Figure 4:**
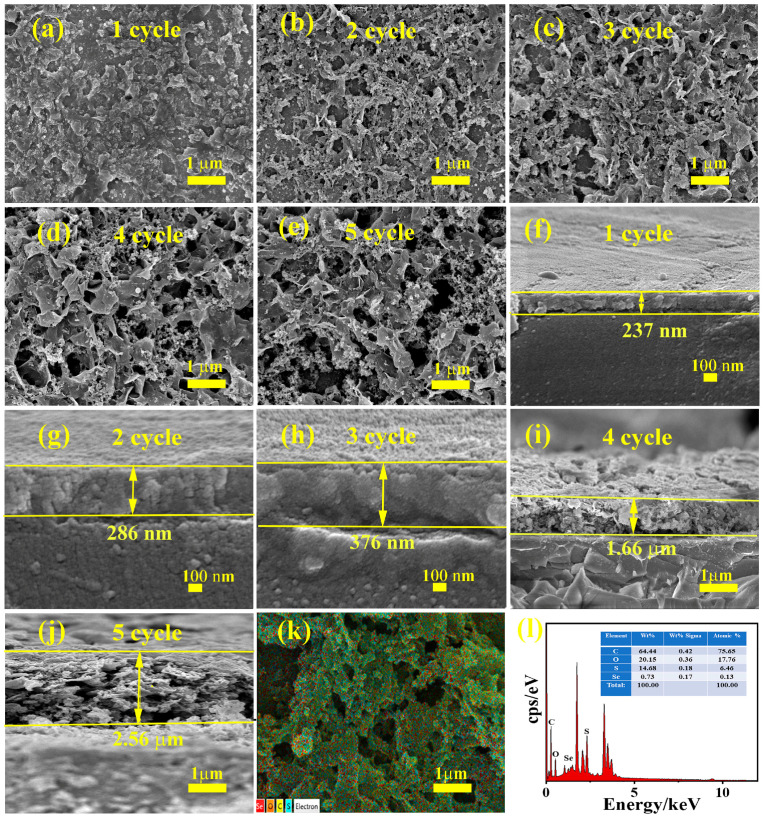
(**a**,**f**) SEM diagram of the top surface and section of the copolymer when the number of polymerization cycles is 1 cycle; (**b**,**g**) SEM diagram of the top surface and section of the copolymer when the number of polymerization cycles is 2 cycles; (**c**,**h**) SEM diagram of the top surface and section of the copolymer when the number of polymerization cycles is 3 cycles; (**d**,**i**) the top surface and cross-section SEM diagram of the copolymer when the number of polymerization cycles is 4 cycles; (**e**,**j**) the top surface and cross-section SEM diagram of the copolymer when the number of polymerization cycles is 5 cycles; (**k**) the mapping diagram of the copolymer; (**l**) the EDS diagram of the copolymer.

Figure 4 indicates the morphologies of P(EDOS-TTh) at different numbers of polymerization cycles when the concentration ratio of EDOS to TTh is 1:1. Figure 4a–i show the top and cross-sectional views of the copolymer from 1 to 5 turns. Figure 4a,f show the top-view and cross-sectional of the copolymer when the number of polymerization cycles is 1, and Figure 4b,g show the top-view and cross-sectional of the copolymer when the number of polymerization cycles is 2. Figure 4c,h show the top-view and cross-sectional of the copolymer when the number of polymerization cycles is 3, and Figure 4d,i show the top-view and cross-sectional of the copolymer when the number of polymerization cycles is 4. Figure 4e,j shows the top view and cross-sectional area of the copolymer when the number of polymerization cycles was 5. The mapping diagram of the copolymer is shown in Figure 4k, and the EDS map of the copolymer is shown in Figure 4l. As shown in Figure 4a,e, the introduction of the TTh unit into the copolymer molecular chain changed the morphology of the polymerized PEDOS membrane; the size of the polymerized compact PEDOS membrane increased from 1 cycle to 5 cycles as the number of polymerization cycles increased. This gradually became a loose fibrous P(EDOS-TTh) copolymer film. The loose fibrous morphology of the copolymer effectively improved the transport, separation, and generation of the photogenerated electron holes in the P-N junction [22]. It is expected that the UV photodetection performance of the P-N junction will improve. Figure 4f,j shows the SEM cross sections of the P(EDOS-TTh) copolymer membranes when the number of polymerization cycles was 1, 2, 3, 4, or 5, respectively. As shown in the figure, as the number of polymerization cycles of the copolymer increased, the thicknesses of the P(EDOS-TTh) copolymer films deposited on the FTO glass surface also increased to 237 nm, 286 nm, 376 nm, 1.66 mm, and 2.56 mm. Figure 4k shows the element mapping diagram of the copolymer when the number of polymerization cycles is 3. It can be seen from the figure that the copolymer contains C, O, S, and Se. Furthermore, these elements were uniformly distributed in P(EDOS-TTh). Figure 4l shows the EDS spectrum of the P(EDOS-TTh) copolymer membrane, with peaks corresponding to C, O, S, and Se occurring at 64.4 wt%, 20.15 wt%, 14.68 wt%, and 0.73 wt%, respectively.

**Figure 5:**
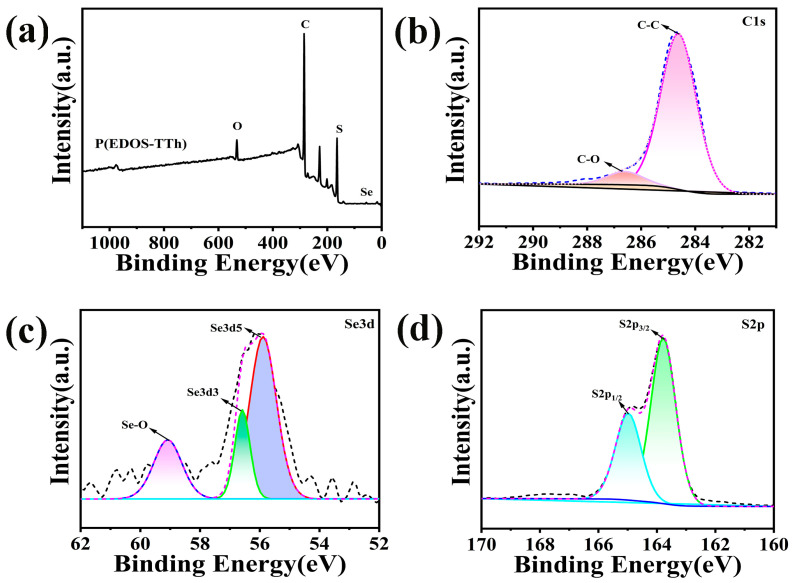
XPS spectra of P(EDOS-TTh): (**a**) survey; (**b**) C 1s; (**c**) Se 3d; (**d**) S 2p.

Figure 5 shows the general XPS spectrum and high-resolution XPS spectra of C, Se, and S in the P(EDOS-TTh) copolymer. As shown in Figure 5a, the copolymer contained C, O, S, and Se. According to the XPS image in Figure 5b, the peaks located at 284.6 and 286.4 eV correspond to the C-C and C-O bonds on the selenophene ring and thiophene ring in the copolymer. In the XPS map of Se 3d in the copolymer (Figure 5c), it can be seen the three peaks with binding energies of 55.8, 56.6, and 59.08 eV, which are attributed to the spin’s cracks of the Se 3d5 and Se 3d3 selenium atoms and Se-O bonds [26]. In the S 2p XPS, the peaks at binding energies of 163.8 and 165.01 eV correspond to the spin cracks of the S atom [27]. XPS analysis revealed that, compared with that in PEDOS, the binding energy of the whole element in the P(EDOS-TTh) copolymer slightly shifted to the direction of higher binding energy, indicating that there are definite bonds and interactions between the atoms in the copolymer.

### 3.3. Performance Analysis of the ZnO/P(EDOS-TTh) Device

**Figure 6:**
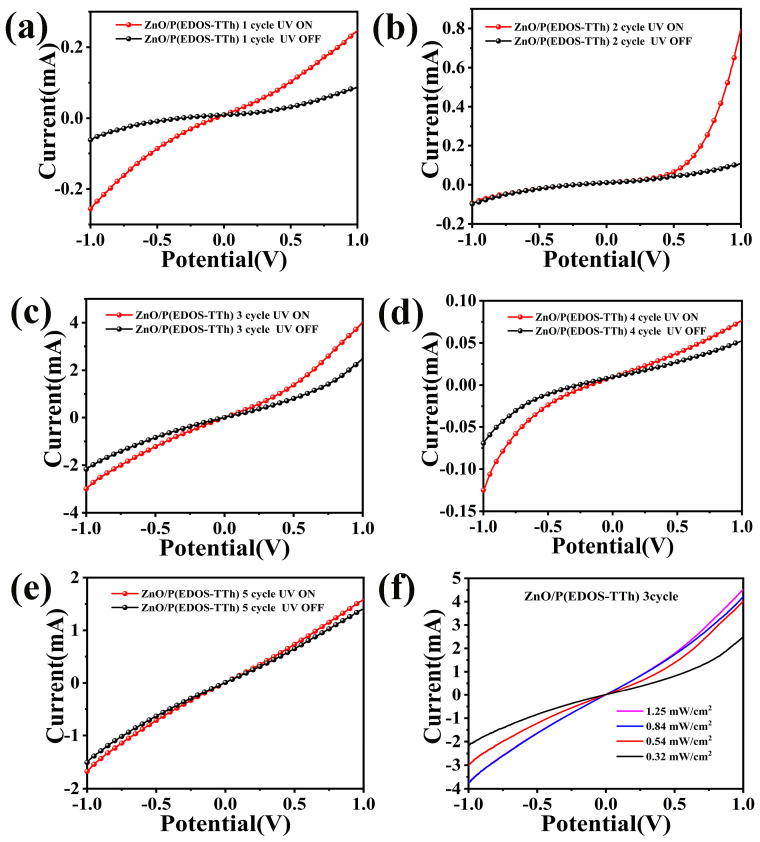
(**a**) The I–V curve of the device assembled by the copolymer when the number of polymerization cycles is 1 cycle. (**b**) The I–V curve of the device assembled by the copolymer when the number of polymerization cycles is 2 cycles. (**c**) The I–V curve of the device assembled by the copolymer when the number of polymerization cycles is 3 cycles. (**d**) The I–V curve of the copolymer-assembled device when the number of polymerization cycles is 4 cycles. (**e**) The I–V of the copolymer assembly at the number of polymerization cycles of 5 cycles. (**f**) The I–V of the UV photodetector assembled by the copolymer at different optical power densities when the number of polymerization cycles is 3 cycles.

Study the effect of different numbers of polymerization cycles on the performance of the assembled UV detectors. ZnO NRs/FTO devices with the same dimensions as the other piece were assembled from 1 to 5 turns of the copolymer with a 1:1 ratio of copolymers, respectively. Figure 6a–e shows the I–V curves of the ZnO/P(EDOS-TTh) P-N heterojunction for different cycles under light and dark conditions. Figure 6a–e show the I–V of the devices assembled with 1~5 cycles of the copolymer. The I–V response curves show that the ZnO/P(EDOS-TTh) P-N heterojunction exhibited asymmetric and nonlinear characteristics, indicating that the assembled ZnO/P(EDOS-TTh) P-N junction had better rectification characteristics [28].

The observed rectification characteristics originate from the P-N junction formed by ZnO and P(EDOS-TTh). When the number of polymerization cycles was 3, the photocurrent density of the ZnO/P(EDOS-TTh) heterojunction device assembled from the copolymer reached 4.05 mA under a bias voltage of −1~+1 V. Figure 6f shows the I–V curves of the ZnO/P(EDOS-TTh) heterojunction assembled with a copolymer when the number of aggregation cycles is 3 under irradiation with UV light at different optical power densities. The heterojunction behaved at 0 V. A clear photoresponse was displayed, and the photocurrent increased with increasing optical power density.

**Figure 7:**
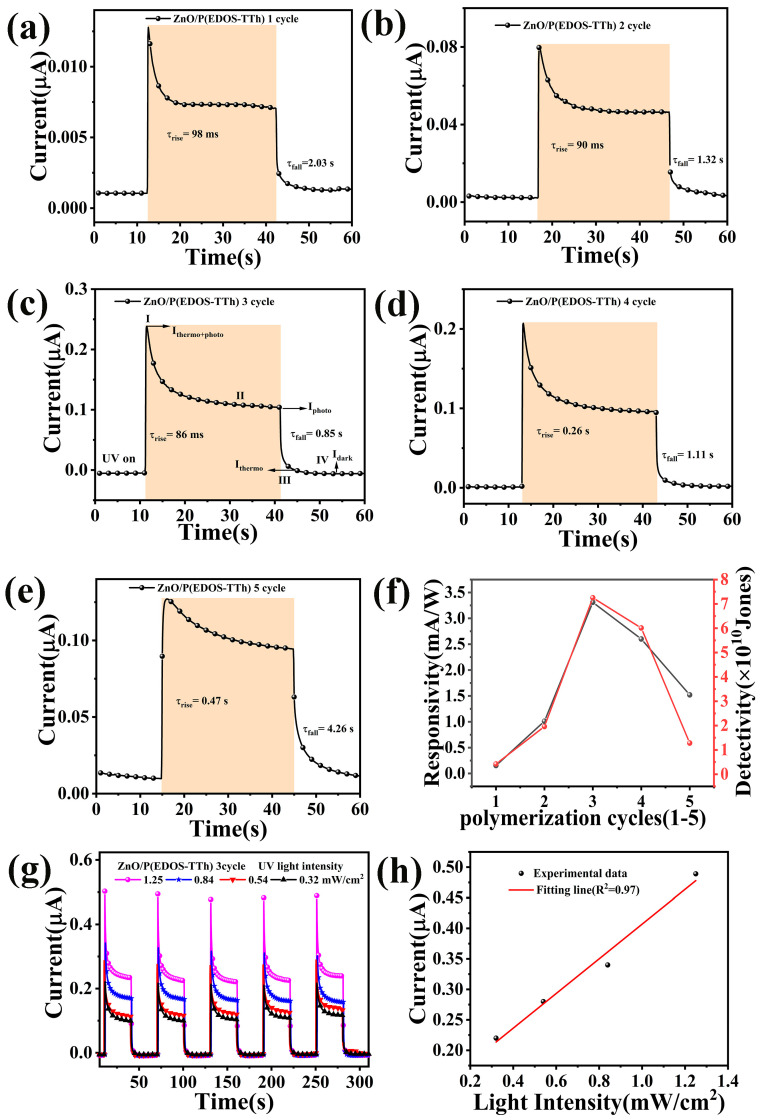
(**a**) the *I-t* of the device assembled by the copolymer when the number of polymerization cycles is 1 cycle; (**b**) the *I-t* of the device assembled by the copolymer when the number of polymerization cycles is 2 cycles; (**c**) the *I-t* of the device assembled by the copolymer when the number of polymerization cycles is 3 cycles; (**d**) the *I-t* of the copolymer assembly P-N junction when the number of polymerization cycles is 4 cycles; (**e**) the *I-t* of the copolymer assembly when the number of polymerization cycles is 5 cycles; (**f**) changes in the responsiveness of devices assembled by copolymers with different polymerization circles; (**g**) *I-t* curves at different optical power densities of copolymer-assembled devices with 3 polymerization cycles; (**h**) relationship between photocurrent and different optical power densities of copolymer-assembled devices with 3 polymerization cycles.

To further study the performance of the heterojunction UV detector, a response performance analysis was performed on the device under a bias voltage of 0 V and an optical power density of 0.32 mW/cm^2^. As illustrated in Figure 7, the photocurrent of the assembled device increased rapidly when UV light was applied, and the current decreased rapidly when the UV light was turned off. Figure 7a–e shows the response time of the devices assembled with copolymer polymerization cycles of 1–5 cycles. The photocurrent of the assembled heterojunction device gradually increases as the number of copolymerizations increases. When the number of aggregation cycles was 3, the highest photocurrent was obtained, and then the photocurrent gradually decreased, first increasing and then decreasing. As shown in Figure 7a–e, the I-t curve is sharp, which is due to the pyroelectric effect generated by the ZnO and copolymer P-N heterojunction [29,30].

Responsiveness (R) and detection rate (D*) are the important parameters for evaluating the UV PDs and represent the formula as follows:

(1) $$R=\frac{I_{ph}-I_{dark}}{P\times S}$$

(2) $$D^{*}=\frac{R}{\sqrt{2eI_{dark}/s}}$$

where P is light power, and S is 0.24 cm^2^ in this study.

Figure 7f shows the relationship diagram between the responsivity of the assembled device and the number of detection and aggregation turns for different numbers of aggregation turns. As shown in the figure, when the number of polymerization cycles was 3, the copolymer-assembled devices exhibited the highest responsivity and detectability. The main performance parameters of the assembled devices are shown in Table 1. A comparison of the performance parameters shows that, when the number of polymerization cycles is three, the ZnO/P(EDOS-TTh) device assembled with a copolymer exhibits excellent UV photodetection performance. The photocurrent reached 0.231 mA under a bias voltage of 0 V, and the values of R and D* reached 3.31 mA/W and 7.25 × 10^10^ Jones, respectively; τ_r_ and τ_f_ are 0.086 s and 0.85 s, respectively.

Figure 7g shows the multicycle *I-t* response curves of the ZnO/P(EDOS-TTh) device assembled with a copolymer under different optical power densities when the number of polymerization cycles is 3. As shown in the figure, as the optical power density of the device increased from 0.32 mW/cm^2^ to 1.25 mW/cm^2^, the photocurrent also increased and showed a linear relationship with the optical power density (Figure 7h). This linear relationship of the UV photodetector reflects that when the number of aggregation cycles is 3, the ZnO/P(EDOS-TTh) device assembled with the copolymer exhibits good UV photodetection performance, indicating that photon absorption by the heterojunction is extremely accurate. The flux is proportional to the efficiency of the photogenerated carriers.

### 3.4. Mechanism Analysis

**Figure 8:**
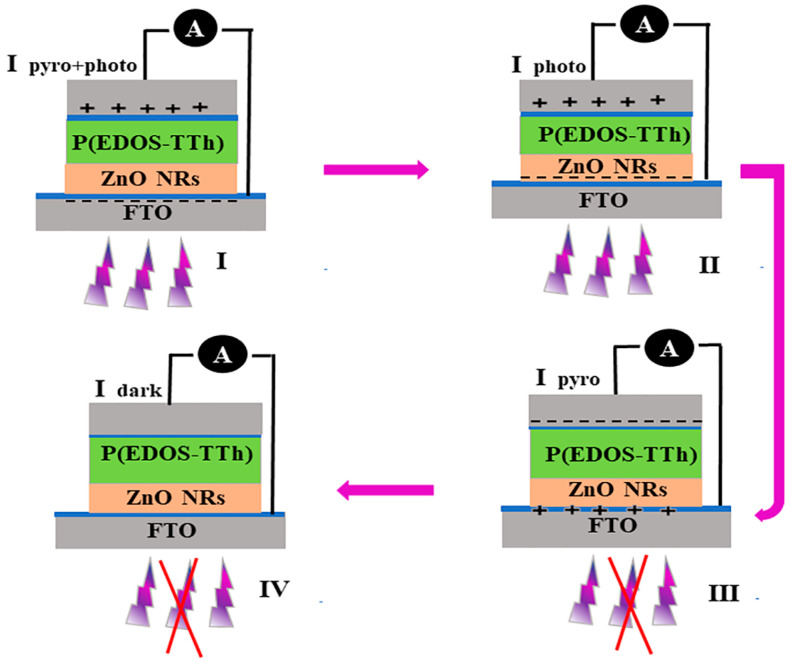
The mechanism of four-stage photocurrent generation in the ZnO/P(EDOS-TTh) hybrid heterojunction.

Figure 8 shows the pyroelectric effect mechanism of the ZnO/P(EDOS-TTh) P-N junction UV detector, including a four-stage current response process [31,32,33]. In the first stage, when the device is irradiated under 365 nm UV light illumination, the temperature of the heterojunction rises instantaneously. Due to thermal polarization, additional carriers are generated in addition to the photovoltaic effect, which is conducive to the generation and operation of photogenerated electron holes. The photocurrent at this stage is expressed as I_pyro+photo_, which is equal to the sum of the current generated by the photovoltaic effect and the thermoelectric effect. In the second stage, due to continuous UV light irradiation, the temperature of the ZnO/P(EDOS-TTh) P-N junction is stable, thus the pyroelectric effect disappears. The current is expressed as the I_photo_, which is only the result of the photovoltaic effect of the ZnO/P(EDOS-TTh) heterojunction.

In the third stage, because the UV light is turned off, the temperature of the device drops instantaneously, forming a thermoelectric effect in the opposite direction, which inhibits the transport of carriers, and the photocurrent also drops instantaneously. This behavior limits the transport of carriers; the photocurrent decreases instantaneously, and the photovoltaic effect is zero, thus generating thermal polarization in the opposite direction. Chemotherapy current (I_pyro_). Finally, in the fourth stage, the thermoelectric effect has disappeared because the transient thermal effect is in a stable state, and the temperature returns to room temperature. The current at this stage is the I_dark_ of the heterojunction.

**Figure 9:**
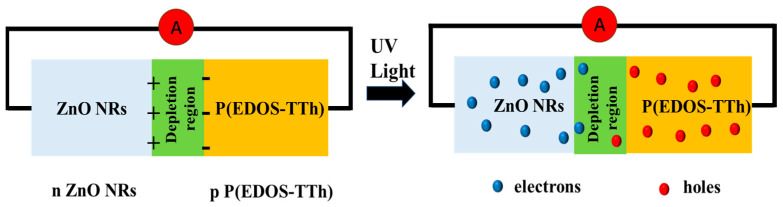
Working principle diagram of ZnO/P(EDOS-TTh) p-n heterojunction.

As illustrated in Figure 9, the working mechanism diagram of the ZnO/P(EDOS-TTh) P-N heterojunction UV photodetector [34]. When N-type ZnO NRs contacted P-type P(EDOS-TTh), there was a good P-N junction at the interface, and a built-in electric field was generated in the junction region. Under UV light irradiation, the built-in electric field generates a certain potential difference, the photogenerated electrons migrate to the side of the ZnO NRs, and the photogenerated holes move to the P(EDOS-TTh) side, facilitating the separation and transport of the photogenerated electron-hole pairs to achieve a self-powered UV light detector. The photogenerated holes migrated from the VB of the ZnO NRs to the HOMO of P(EDOS-TTh) and then migrated to the FTO electrode [35]. In addition, the photogenerated electrons are transferred from the LUMO of P(EDOS-TTh) to the CB of the ZnO NRs and subsequently to the FTO. The excellent matching of the energy level between p-type P(EDOS-TTh) and n-type ZnO NRs promotes the transfer of charges at the P-N junction interface and decreases the recombination of photogenerated electron-holes, thus improving the ZnO/P(EDOS-TTh) performance of the P-N junction UV photodetector.

Table 2 shows the comparison of the performance parameters of the ZnO/P(EDOS-TTh) P-N heterojunction assembled in this study with the main performance parameters of recently reported detectors. The results show that, compared with the ZnO/PANI heterojunction UV detector, the constructed ZnO/P(EDOS-TTh) P-N junction self-powered device has a certain advantage in responsivity, which is different from that of PTAA (55 nm)/GaN. The device constructed has certain advantages in terms of detectability and response time. The D* and R of the ZnO/P(EDOS-TTh) P-N junction device were 7.25 × 10^10^ Jones and 3.31 mA/W, and the τ_r_/τ_f_ was 0.086/0.85 s. This is due to the formation of a higher-quality p-n junction between the P-type P(EDOS-TTh) and the N-type ZnO NRs and the generation of a built-in electric field. This is conducive to the transport and separation of photogenerated electron-holes; the reduced recombination rate of the photogenerated carriers in the junction thus improves the optoelectronic performance of the heterojunction.

## 4. Conclusions

In this study, a P(EDOS-TTh) copolymer was prepared via electrochemical deposition by using EDOS and TTh as comonomers, and a UV photodetector was assembled face-to-face with ZnO NRs. The number of electrochemical aggregation cycles was controlled; thus, the interfacial contact area between the ZnO NRs and P(EDOS-TTh) in the ZnO/P(EDOS-TTh) heterojunction was investigated to determine the effect of the copolymer on the properties of the P-N junction UV photodetector. The results implied that there was an increase in the number of TTh structural units in the copolymer chain, and the compact morphology of the copolymer membrane changed to that of the loose and porous P(EDOS-TTh) copolymer membrane. This loose and porous morphology is conducive to the transport and separation of photogenerated electron-holes, thereby improving the UV photodetection performance of the ZnO/P(EDOS-TTh) heterojunction. When the number of aggregation cycles was 3, the contact between the n-type ZnO NRs and the p-type copolymer P(EDOS-TTh) was the greatest, and the materials formed a good p-n junction and strong built-in electric field, indicating high-performance self-powered UV photodetection. When the R of the heterojunction reached 3.31 mA/W and D* reached 7.25 × 10^10^ Jones, the response time was shortened to 0.086 s and 0.85 s, and good self-powered UV light photodetection was realized with the ZnO NRs.

## Figures and Tables

**Table 1:** Comparison of parameters of devices assembled in different polymerization cycles.

Device	Wavelength (nm)	*I*_ph_/*I*_dark_ (mA)	R (mA/W)	D* (Jone)	τ_r_/τ_f_ (s)
ZnO/P(EDOS-TTh)-1 cycle	365	0.012/0.001	0.154	4.2 × 10^9^	0.098/2.03
ZnO/P(EDOS-TTh)-2 cycles	365	0.08/0.002	1.01	1.96 × 10^10^	0.090/1.32
ZnO/P(EDOS-TTh)-3 cycles	365	0.238/−0.005	3.31	7.25 × 10^10^	0.086/0.85
ZnO/P(EDOS-TTh)-4 cycles	365	0.207/0.001	2.60	6.01 × 10^10^	0.26 s/1.11
ZnO/P(EDOS-TTh)-5 cycles	365	0.127/0.01	1.52	1.28 × 10^10^	0.47 s/4.26

**Table 2:** Recent reports on the key parameters of self-powered photodetectors.

Device	Wavelength (nm)	R (mA/W)	τ_r_/τ_f_ (s)	D* (Jones)	Refs.
ZnO/PANI	355	0.56	0.11/1.45 m	-	[36]
ZnAl:LDH-PDMS	355	148	-	(2.5 ± 0.2) × 10^12^	[37]
GaN/PANI	361	69	1.1/1.8 m	8.45 × 10^13^	[38]
ZnO/PVK	350	9.96	1.5/6	-	[39]
TiO_2_/PC71BM/PEDOT:PSS	350	33	60/1 μ	1.6 × 10^11^	[40]
PTAA(55 nm)/GaN	325	268	14/23	2.155 × 10^9^	[41]
TiO_2_ NRs/P(TTh-co-EDOT)	365	2.52	0.256/0.427	3.413 × 10^10^	[42]
ZnO NRs/P(EDOS-TTh)	365	3.31	0.086/0.85	7.25 × 10^10^	This work

## Data Availability

Data are contained within the article and Supplementary Materials.

## Supplementary Materials

The following supporting information can be downloaded at: https://www.mdpi.com/article/10.3390/nano14080720/s1, Scheme S1: The synthetic route of EDOS monomer; Figure S1: 1H-NMR spectra of 3,4-dimethoxyselenophene and EDOS in CDCl3.

## References

[1] Perez-Tomas A., Chikoidze E., Dumont Y., Jennings M.R., Russell S.O., Vales-Castro P., Catalan G., Lira-Cantu M., Ton–That C., Teherani F.H. (2019). Giant bulk photovoltaic effect in solar cell architectures with ultra-wide bandgap Ga_2_O_3_ transparent conducting electrodes. Mater. Today Energy.

[2] Zheng Y., Li Y., Tang X., Wang W., Li G. (2020). A Self-powered High-performance UV Photodetector Based on Core-Shell GaN/MoO_3_-x Nanorod Array Heterojunction. Adv. Opt. Mater..

[3] Fattah M.F.A., Khan A.A., Anabestani H., Rana M.M., Rassel S., Therrien J., Ban D. (2021). Sensing of ultraviolet light: A transition from conventional to self-powered photodetector. Nanoscale.

[4] Hu L., Zhu L., He H., Guo Y., Pan G., Jiang J., Jin Y., Sun L., Ye Z. (2013). Colloidal Chemically Fabricated ZnO: Cu-based Photodetector with Extended UV-visible Detection Waveband. Nanoscale.

[5] Wang M., Zhang J., Xin Q., Yi L., Guo Z., Wang Y., Song A. (2022). Self-powered UV photodetectors and imaging arrays based on NiO/IGZO heterojunctions fabricated at room temperature. Opt. Express.

[6] Briseno A.L., Holcombe T.W., Boukai A.I., Garnett E.C., Shelton S.W., Fréchet J.J.M., Yang P. (2010). Oligo- and Polythiophene/ZnO Hybrid Nanowire Solar Cells. Nano Lett..

[7] Chandiran A.K., Abdi-Jalebi M., Nazeeruddin M.K., Gratzel M. (2014). Analysis of Electron Transfer Properties of ZnO and TiO_2_ Photoanodes for Dye-Sensitized Solar Cells. ACS Nano.

[8] Bera A., Peng H.Y., Lourembam J., Shen Y.D., Sun X.W., Wu T. (2013). A Versatile Light-Switchable Nanorod Memory: Wurtzite ZnO on Perovskite SrTiO_3_. Adv. Funct. Mater..

[9] Liu X.Y., Shan C.X., Jiao C., Wang S.P., Zhao H.F., Shen D.Z. (2014). Pure Ultraviolet Emission from ZnO Nanowire-Based p-n Heterostructures. Opt. Lett..

[10] Soci C., Zhang A., Xiang B., Dayeh S.A., Aplin D.P., Park J., Bao X.Y., Lo Y.H., Wang D. (2007). ZnO Nanowire UV Photodetectors with High Internal Gain. Nano Lett..

[11] Das S.N., Moon K.J., Kar J.P., Choi J.H., Xiong J., Lee T.I., Myoung J.M. (2010). ZnO Single Nanowire-Based UV Detectors. Appl. Phys. Lett..

[12] Jin Y.Z., Wang J.P., Sun B.Q., Blakesley J.C., Greenham N.C. (2008). Solution-Processed Ultraviolet Photodetedtors Based on Colloidal ZnO Nanoparticles. Nano Lett..

[13] Ghosh T., Basak D. (2010). Highly Efficient Ultraviolet Photodetection in Nanocolumnar RF Sputtered ZnO Films: A Comparison between Sputtered, Sol-Gel and Aqueous Chemically Grown Nanostructures. Nanotechnology.

[14] Li Q., Huang J., Meng J.P., Li Z. (2022). Enhanced performance of a self-powered ZnO photodetector by coupling LSPR-inspired pyro-pho totronic effect and piezo-phototronic effect. Adv. Opt. Mater..

[15] Chen J., Xu B., Ma H., Qi R., Bai W., Yue F., Yang P., Chen Y., Chu J., Sun L. (2024). Element Diffusion Induced Carrier Transport Enhancement in High-Performance CZTSSe Self-Powered Photodetector. Small.

[16] Zhang J., Sa X., Li S., Zhai J. (2024). All-Solution-Processed InGaO/PbI_2_ Heterojunction for Self-Powered Omnidirectional Near-Ultraviolet Photodetection and Imaging. Adv. Opt. Mater..

[17] Rana A.K., Kumar M., Ban D.K., Wong C.P., Yi J., Kim J. (2019). Enhancement in performance of transparent p-NiO/n-ZnO heterojunction ultrafast self-powered photodetector via pyro-phototronic effect. Adv. Electron. Mater..

[18] Jiang W., Zheng T., Wu B., Jiao H., Wang X., Chen Y., Zhang X., Peng M., Wang H., Lin T. (2020). A versatile photodetector assisted by photovoltaic and bolometric effects. Light. Sci. Appl..

[19] Hu L., Qiao W., Han J., Zhou X., Wang C., Ma D., Wang Z.Y., Li Y. (2017). Naphthalene diimide-diketopyrrolopyrrole copolymers as non-fullerene acceptors for use in bulk-heterojunction all-polymer UV-NIR photodetectors. Polym. Chem..

[20] Tong S., Yuan J., Zhang C., Wang C., Liu B., Shen J., Xia H., Zou Y., Xie H., Sun J. (2018). Large-scale roll-to-roll printed, flexible and stable organic bulk heterojunction photodetector. npj Flex. Electron..

[21] Li S., Deng X., Feng L., Miao X., Tang K., Li Q., Li Z. (2016). Copolymers of carbazole and phenazine derivatives: Minor structural modification, but totally different photodetector performance. Polym. Chem..

[22] Pickett A., Mohapatra A., Laudari A., Khanra S., Ram T., Patil S., Guha S. (2017). Hybrid ZnO-organic semiconductor interfaces in photodetectors: A comparison of two near-infrared donor-acceptor copolymers. Org. Electron..

[23] Dai Y., Li W., Zhao R., Huang Q., Xu N., Yuan F., Zhang C. (2019). Quadruple thiophene based electrochromic electrodeposited film as high performance hybrid energy storage system. Electrochim. Acta.

[24] Ahmed M.S., Jeong H., You J.M., Jeon S. (2012). Synthesis and characterization of an electrochromic copolymer based on 2,2′:5′,2″-terthiophene and 3,4-ethylenedioxythiophene. Appl. Nanosci..

[25] Kadir A., Jamal R., Abdiryim T., Sawut N., Che Y., Helil Z., Zhang H. (2021). Electrochemical sensor formed from poly(3,4-ethylenedioxyselenophene) and nitrogen-doped graphene composite for dopamine detection. RSC Adv..

[26] Zhu Z., Li Z., Xiong X., Hu X., Wang X., Li N., Jin T., Chen Y. (2022). ZnO/ZnSe heterojunction nanocomposites with oxygen vacancies for acetone sensing. J. Alloys Compd..

[27] Feng W., Wan A.S., Garfunkel E. (2013). Interfacial Bonding and Morphological Control of Electropolymerized Polythiophene Films on ZnO. J. Phys. Chem. C.

[28] Mojtabavi E.A., Nasirian S. (2019). Flexible self-powered ultraviolet-visible photodetector based on polyaniline- titanium dioxide heterostructures: The study of the rearrangement of layers. Appl. Surf. Sci..

[29] Jiang W., Zhao T., Liu H., Jia R., Niu D., Chen B., Shi Y., Yin L., Lu B. (2018). Laminated pyroelectric generator with spin coated transparent poly(3,4-ethylenedioxythiophene) polystyrene sulfonate (PEDOT:PSS) electrodes for a flexible self-powered stimulator. RSC Adv..

[30] Fan Z., Ouyang J. (2019). Thermoelectric Properties of PEDOT:PSS. Adv. Electron. Mater..

[31] Feng Y., Zhang Y., Wang Y., Wang Z. (2018). Frequency response characteristics of pyroelectric effect in p-n junction UV detectors. Nano Energy.

[32] Serrano-Claumarchirant J.F., Culebras M., Muñoz-Espí R., Cantarero A., Gómez C.M., Collins M.N. (2019). PEDOT Thin Films with n-Type Thermopower. ACS Appl. Energy Mater..

[33] Lee J., Kim H.J., Ko Y.J., Baek J.Y., Shin G., Jeon J.G., Lee J.H., Kim J.H., Jung J.H., Kang T.J. (2022). Enhanced pyroelectric conversion of thermal radiation energy: Energy harvesting and non-contact proximity sensor. Nano Energy.

[34] Luo G., Yang X., Long Y., Li W., Yang Y., Luo S. (2022). Enhanced performance of self-powered ultraviolet photodetectors coupled with the photovoltaic-pyroelectric effect based on ZnO/CuBO_2_ core-shell nanorod arrays. J. Alloys Compd..

[35] Ding M., Zhao D., Yao B., Li Z., Xu X. (2015). Ultraviolet photodetector based on heterojunction of n-ZnO microwire/p-GaN film. RSC Adv..

[36] Chen Y., Su L., Jiang M., Fang X. (2022). Switch type PANI/ZnO core-shell microwire heterojunction for UV photodetection. J. Mater. Sci. Technol..

[37] Thomas A.M., Yoon C., Ippili S., Jella V., Yang T.Y., Yoon G., Yoon S.G. (2021). High-Performance Flexible Ultraviolet Photodetectors Based on Facilely Synthesized Ecofriendly ZnAl:LDH Nanosheets. ACS Appl. Mater. Interfaces.

[38] Sun Y., Song W., Gao F., Wang X., Luo X., Guo J., Zhang B., Shi J., Cheng C., Liu Q. (2020). In Situ Conformal Coating of Polyaniline on GaN Microwires for Ultrafast, Self-Driven Heterojunction Ultraviolet Photodetectors. ACS Appl. Mater. Interfaces.

[39] Dong Y., Zou Y., Song J., Zhu Z., Li J., Zeng H. (2016). Self-powered fiber-shaped wearable omnidirectional photodetectors. Nano Energy.

[40] Yan T., Cai S., Hu Z., Li Z., Fang X. (2021). Ultrafast Speed, Dark Current Suppression, and Self-Powered Enhancement in TiO_2_-Based Ultraviolet Photodetectors by Organic Layers and Ag Nanowires Regulation. J. Phys. Chem. Lett..

[41] Chen F., Deng C., Wang X., Liu C., Liu Q., Zou C., Wu G., Zhao Z., Chen K., Gao F. (2022). Enhanced Photoresponse Performance of Self-Powered PTAA/GaN Microwire Heterojunction Ultraviolet Photodetector Based on Piezo-Phototronic Effect. Adv. Mater. Interfaces.

[42] Tang X., Zhang H., Jamal R., Abdurexit A., Serkjan N., Xie S., Liu Y., Abdiryim T. (2024). High performance self-powered ultraviolet photodetectors based on P(TTh-co-EDOT) copolymer sensitized TiO_2_ NRs. Surf. Interfaces.

