# Supplementary material for: Self-Powered UV Photodetector Construction of the P(EDOS-TTh) Copolymer-Modified ZnO Nanoarray
Source: Nanomaterials (Basel). 2024 Apr 20;14(8):720. doi: 10.3390/nano14080720 (PMC11053458; doi:10.3390/nano14080720)
Supplement: Supplementary file 1 [file nanomaterials-14-00720-s001.zip › nanomaterials-2903704-supplementary.pdf]

Supplementary Materials

Self-powered UV photodetector Construction of the P(EDOS-TTh) Copolymer-Modified ZnO Nanoarray

Aygul Kadir[a,b], Tursun Abdiryim[a*], Xiong Liu[a], Ruxangul Jamal[b], Yaolong Zhang[a]

1 State Key Laboratory of Chemistry and Utilization of Carbon Based Energy Resources, Key Laboratory of Advanced Functional Materials, College of Chemistry, Xinjiang University, Urumqi 830046, China; aygul419@sina.com (A.K.); liuxiong@xju.edu.cn (X.L.); zyl1754915507@sina.com (Y.Z.)

2 Key Laboratory of Petroleum and Gas Fine Chemicals, Educational Ministry of China, College of Chemical Engineering, Xinjiang University, Urumqi 830046, China; jruxangul@xju.edu.cn

* Correspondence: tursunabdir@sina.com.cn; Tel./Fax: +86-099-1858-2809

Preparation of the ZnO NRs

According to previously reported literature[1], ZnO NRs were grown on FTO conducting glass by hydrothermal method. The fluorine-doped tin oxide (FTO) Glass substrates were initially ultrasonic cleaned with acetone, ethanol and deionized water,

successively, and then blown dry with dry air. ZnO seed layer was firstly deposited on the fluorine-doped tin oxide (FTO) glass by spin-coating process. Preparation of zinc oxide solvent: dissolve 0.5488 g zinc dihydroacetate in 50 ml ethanol. At the 60°C the amount of eththanolamine (about 0.15 ml) with zinc is added to the above solution and stirred for 2 h to stabilize the transparent solvent. An aqueous solution for ZnO growth was prepared with 25 mM hexamethylenetetramine and 25 mM zinc nitrate. A piece of FTO substrate with the ZnO seed layer was placed at an angle against the wall of the autoclave with the conducting side facing down. The autoclave was sealed and placed in an oven at 95℃ for 4 h before the sample was rinsed with deionized water.

Synthesis of EDOS

2,3-dimethoxy-1,3-butadiene was synthesized according to a previously reported method[2].

EDOS was synthesized according to a previously reported method[3]. First prepared $SeCl_2$, SO_2Cl_2 and selenium powder were as reactants, and n-hexane as solvent. A solution of freshly-prepared $SeCl_2$ in hexanes was added to a well-stirred mixture of 2,3-dimethoxy-1,3-butadiene (24.0 g, 210 mmol) and CH_3COONa (8.2 g, 100 mmol) (as the buffering agent) in hexanes (250 mL) (as the solvent) at –78 °C under an inert atmosphere. The resulting yellowish solution was further stirred for 1 h at -78 °C and the reaction mixture was warmed to room temperature stirred for another 4 h. The reaction mixture was filtered and washed with n-hexane. The residue was concentrated to provide a brown oil that was purified by recrystallization in hexanes at low temperature to furnish a white crystalline solid. 1H NMR (400 MHz, $CDCl_3$): δ

(ppm) = 6.55 (s, 2H), 3.85 (s, 6H).

A solution of 3,4-dimethoxyselenophene (1 g, 5.24 mmol), with 6 equivalents of ethylene glycol (2 g, 32.41 mmol) and a *p*-toluene sulfonic acid (160 mg) (as catalytic agent) in dry toluene (150 mL) was stirred for 12 h at 50 – 55 °C. Toluene was removed under reduced pressure, and the residue was diluted with water (100 mL). The mixture was extracted with dichloromethane (3 x 50 mL). The combined organic layers were washed with dilute NaCl solution and brine and then Spinning solvent to concentrated. Purification of the crude residue by silica gel chromatography gave 3,4-ethylenedioxyselenophene as a colorless liquid. 1H NMR (400 MHz, CDCl3): δ (ppm)= 6.79 (s, 2H), 4.17 (s, 4H).

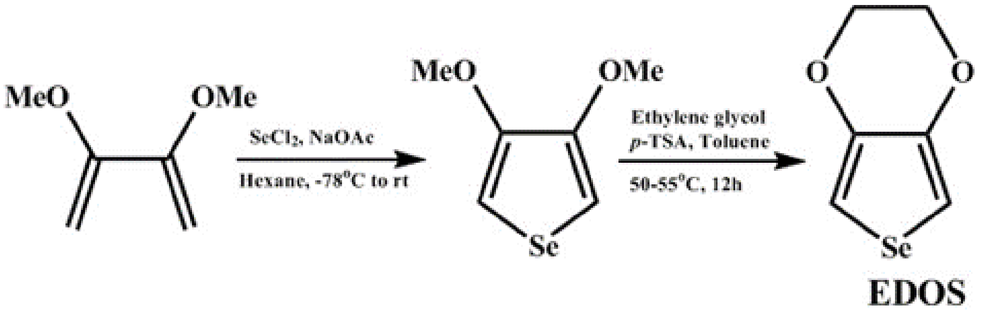

Scheme S1. The synthetic route of EDOS monomer

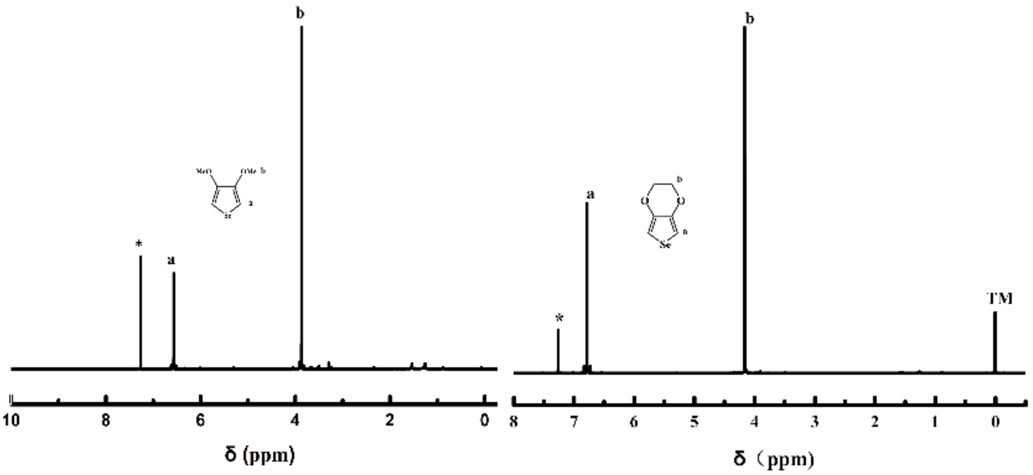

Fig. S1 1H-NMR spectra of 3,4-dimethoxyselenophene and EDOS in $CDCl_3$.
